# Supplementary material for: Animal welfare with Chinese characteristics: Chinese poultry producers’ perceptions of, and attitudes towards, animal welfare
Source: PLoS One. 2024 Jul 18;19(7):e0307061. doi: 10.1371/journal.pone.0307061 (PMC11257329; doi:10.1371/journal.pone.0307061)
Supplement: S1 Appendix — (DOCX) [file pone.0307061.s001.docx]

**Interview guide for cage egg producers**

1. Which province is your farm in?
2. What is the farm size (number of birds)?
3. What is your job position on the farm?
4. What production system do you use?
5. What led you to choose this production system?
6. What are the benefits of using this production system?
7. What are the disadvantages of using this production system?
8. How do you feel about using this system?
9. Have you heard of cage-free systems?
10. What do you think cage-free systems are?
11. What do you think of cage-free systems compared to cage systems?
12. What do you think are the reasons for some producers to choose cage-free systems?
13. If you were to use cage-free systems, what would you consider before you start？
14. Do you need support to change to cage-free systems?
15. What kind of help do you need to change to cage-free systems?
16. What can encourage more cage producers to try cage-free systems?
17. How likely will you try cage-free systems in the next five years?
18. What do you think of hens?
19. What do you think animal welfare is?
20. What impact do cages have on animal welfare?
21. What impact do cage-free systems have on animal welfare?
22. How important do you think animal welfare is in farming?

**Interview Guide For cage-free egg producers**

1. Which province is your farm in?
2. What is the farm size (number of birds)?
3. What is your job position on the farm?
4. What production system do you use?
5. Did you use this production system since you set up this farm?
6. What led you to choose/change to this production system?
7. What are the benefits of using this production system?
8. What are the disadvantages of using this production system?
9. How do you feel about using this system?
10. What were your biggest difficulties when running your farm?
11. How did you overcome those challenges?
12. Do you think you need support in your farm operation?
13. What kind of support?
14. Who can provide such support?
15. Have you heard of cage-free systems?
16. What do you think cage-free systems are?
17. Have you observed any cage farms that want to try cage-free systems?
18. What are your suggestions for those producers who want to try cage-free systems?
19. What would help those producers to change to cage-free systems?
20. What can encourage more producers to try cage-free systems?
21. Will you still run your farm in the next five years? How likely are you going to expand it? How likely are you going to try other cage-free systems?
22. What do you think of hens?
23. What do you think animal welfare is?
24. What impact do cages have on animal welfare?
25. What impact do cage-free systems have on animal welfare?
26. How important do you think animal welfare is in farming?
