# Supplementary material for: Animal welfare with Chinese characteristics: Chinese poultry producers’ perceptions of, and attitudes towards, animal welfare
Source: PLoS One. 2024 Jul 18;19(7):e0307061. doi: 10.1371/journal.pone.0307061 (PMC11257329; doi:10.1371/journal.pone.0307061)
Supplement: S2 Appendix — (DOCX) [file pone.0307061.s002.docx]

**Interview guide for cage broiler producers**

1. Which province is your farm in?
2. What is the farm size (number of birds)?
3. What is your job position on the farm?
4. What production system do you use?
5. Did you use this system since you set up this farm?
6. What led you to choose/change to this production system?
7. What are other production systems in broiler farming?
8. What system is more prevalent in broiler farming?
9. What are the benefits of using this production system?
10. What are the disadvantages of using this production system?
11. How do you feel about using this system?
12. What do you think of cage systems compared to floor-based systems?
13. What are the reasons why some producers change to cage systems?
14. Why do you think some producers change to floor-based systems?
15. How likely will you use floor-based systems in the next five years?
16. What do you think of broilers?
17. What do you think animal welfare is?
18. What impact do cages have on animal welfare?
19. What impact do floor-based systems have on animal welfare?
20. How important do you think animal welfare is in broiler farming?

**Interview guide for floor-based broiler producers**

1. Which province is your farm in?
2. What is the farm size (number of birds)?
3. What is your job position on the farm?
4. What production system do you use?
5. Have you used a floor-based system since setting up this farm?
6. What led you to choose/change to this production system?
7. What are other production systems in broiler farming?
8. What system is more prevalent in broiler farming?
9. What are the benefits of using a floor-based production system?
10. What are the disadvantages of using a floor-based production system?
11. How do you feel about using this system?
12. What do you think of cage systems?
13. What were your biggest challenges when running your farm?
14. How did you overcome those challenges?
15. Do you think you will still be using floor-based systems in the next five years? How likely are you going to expand it?
16. Thinking of your own business, what would help you to operate with a floor-based system?
17. Thinking of the industry, what would help producers to stay with floor-based systems?
18. What do you think of broilers?
19. What do you think animal welfare is?
20. What impact do cages have on animal welfare?
21. What impact do floor-based systems have on animal welfare?
22. How important do you think animal welfare is in broiler farming?
